# Loop-mediated isothermal amplification: rapid detection of *Angiostrongylus cantonensis *infection in *Pomacea canaliculata*

**DOI:** 10.1186/1756-3305-4-204

**Published:** 2011-10-25

**Authors:** Rui Chen, QunBo Tong, Yi Zhang, Di Lou, QingMing Kong, Shan Lv, MingMing Zhuo, LiYong Wen, ShaoHong Lu

**Affiliations:** 1 Institute of Parasitology, Zhejiang Academy of Medical Sciences, Hangzhou, People's Republic of China; 2 National Institute of Parasitic Diseases, Chinese Center for Disease Control and Prevention, Shanghai, People's Republic of China

## Abstract

**Background:**

*Angiostrongylus cantonensis *is a zoonotic parasite that causes eosinophilic meningitis in humans. The most common source of infection with *A. cantonensis *is the consumption of raw or undercooked mollusks (e.g., snails and slugs) harbouring infectious third-stage larvae (L_3_). However, the parasite is difficult to identify in snails. The purpose of this study was to develop a quick, simple molecular method to survey for *A. cantonensis *in intermediate host snails.

**Findings:**

We used a loop-mediated isothermal amplification (LAMP) assay, which was performed using *Bst *DNA polymerase. Reactions amplified the *A. cantonensis *18S rRNA gene and demonstrated high sensitivity; as little as 1 fg of DNA was detected in the samples. Furthermore, no cross-reactivity was found with other parasites such as *Toxoplasma gondii, Plasmodium falciparum, Schistosoma japonicum, Clonorchis sinensis, Paragonimus westermani *and *Anisakis*. *Pomacea canaliculata *snails were exposed to *A. cantonensis *first-stage larvae (L_1_) in the laboratory, and L_3 _were observed in the snails thirty-five days after infection. All nine samples were positive as determined by the LAMP assay for *A. cantonensis*, which was identified as positive by using PCR and microscopy, this demonstrates that LAMP is sensitive and effective for diagnosis.

**Conclusions:**

LAMP is an appropriate diagnostic method for the routine identification of *A. cantonensis *within its intermediate host snail *P. canaliculata *because of its simplicity, sensitivity, and specificity. It holds great promise as a useful monitoring tool for *A. cantonensis *in endemic regions.

## Findings

*Angiostrongylus cantonensis *can be found in the lungs and arteries of insectivores, rodents, canines and felines [1-6]. They are prevalent in the Pacific islands and Southeast Asia, and are the most common cause of eosinophilic meningitis in humans in areas where the parasite is endemic [7]. The definitive hosts of *A. cantonensis *are various species of rats. Modes of transmission of this parasite include ingestion of raw or undercooked snails and fresh leafy vegetables contaminated by infective third-stage larvae (L_3_) [8]. First-stage larvae (L_1_) of *A. cantonensis *grow to infective L_3 _in intermediate host snails. This disease is difficult to detect because of the long incubation period in patients and few diagnostic symptoms. Thus, in order to control *A. cantonensis*, efforts should be directed towards building a surveillance system for the intermediate host snails of this parasite.

Many species of snails can serve as intermediate hosts for *A. cantonensis*. The first national survey pertaining to *A. cantonensis *and its definitive and intermediate hosts in the People's Republic of China (P.R.China) was implemented in 2006-2007 [9], and the results showed that the endemicity of *A. cantonensis *is primarily attributable to *P. canaliculata *and *Achatina fulica*, which were imported into P.R.China in 1981 [10,11] and 1931 [12,13], respectively, and have rapidly extended their geographic ranges. Indeed, these two snails are now listed as invasive species by the Chinese government. *P. canaliculata *played an important role in a recent angiostrongyliasis outbreak in P.R.China [14-17].

Current surveying of *A. cantonensis *intermediate host snails depends mainly on microscopic examination. However, the parasite is difficult to identify and it is often overlooked if the infective load is low. Furthermore, microscopy is laborious and time-consuming and is not suitable for large-scale surveys. Application of polymerase chain reaction (PCR) is considered a more accurate and practical diagnostic method [18]; however, despite its excellent sensitivity and specificity, a PCR approach requires special equipment and is not suitable for field application. With this in mind, we propose a novel detection method based on loop-mediated isothermal amplification (LAMP).

LAMP is a novel method for the rapid amplification of DNA. Its advantages include rapidity and minimal equipment requirements. *Bst *DNA polymerase can synthesize a new strand of DNA, while simultaneously displacing the complementary strand, thereby enabling DNA amplification at a single temperature with a single enzyme. Four primers are required for the LAMP reaction: FIP, BIP, F3 and B3. F3 and B3 contribute to the formation of a stem-loop structure, while the other two primers, FIP and BIP, which are complementary to the inner sequence of the stem-loop structure, are used to amplify the target sequence. Thus, using four amplification primers provides higher specificity to the reaction than conventional PCR method. Another advantage of LAMP is that amplification from stem-loop structures leads to the accumulation of a large amount of products of varying lengths, which make detection of amplified DNA much easier, the result can easily be seen by the naked eye.

Recently, the applicability of LAMP for the detection of parasites such as *Trypanosoma, Babesia *and *Plasmodium *has been demonstrated [19-26]. However, a LAMP application for survey of the intermediate host snails of *A. cantonensis *has not been developed. This report describes the establishment of a LAMP assay for the sensitive and specific detection of *A. cantonensis *in snails.

### Ethics statement

The institutional ethics committee of Zhejiang Academy of Medical Sciences in Hangzhou approved this study. Animal experiments were carried out in adherence to institutional guidelines for animal husbandry.

### Parasite preparation and experimental infection

An *A. cantonensis *animal infection model had been established in the lab [27,28]. A total of five Sprague-Dawley (SD) rats were infected by intragastric injection of 50-80 L_3_. Thirty-five days post-infection, fresh faeces (i.e., not older than 12 h) were collected from infected rats and the L_1 _larvae were isolated by the Baermann technique [5]. Eggs of *P. canaliculata *were cultured in the laboratory to make certain that developing snails remained uninfected throughout the study. A total of nine adult snails were fasted for 48 h, and then exposed to a 200-ml suspension of 40,000 L_1 _in a dish (radius = 8.5 cm, height = 6.5 cm) for 12 h at room temperature. Subsequently, the snails were transferred to aquariums with clean water maintained at a temperature of 21 ± 1°C and were fed a mixed diet consisting of vegetables and dried fish food. For the examination of *P. canaliculata*, a recently developed method relying on specific lung tissue features of this species was employed [28,29]. In brief, the lungs were separated from the snail body and opened. The nodules containing *A. cantonensis *larvae were then directly observed under a microscope. The above establishment of the infection model and experimental snail infection was conducted at the National Institute of Parasitic Diseases, Chinese Center for Disease Control and Prevention in Shanghai.

### DNA extraction

Genomic DNA from *A. cantonensis *larvae, *T. gondii, P. falciparum*, adult worms of *S. japonicum, C. sinensis, P. westermani *and *Anisakis *larvae was extracted by using a QIAamp DNA mini kit (QIAGEN Inc; Valencia, CA, USA) and the concentration of DNA was measured using a NanoDrop 2000 spectrophotometer (Thermo Scientific; Wilmington, DE, USA). The lungs of nine infected and nine uninfected *P. canaliculata *snails were separated from the snail body and DNA was extracted for the LAMP and PCR assay. DNA derived from *T. gondii, P. falciparum, S. japonicum, C. sinensis, P. westermani *and *Anisakis *was used to demonstrate the specificity of the LAMP assay. The DNA was finally eluted in a 60-μl volume of elution buffer and stored at -20°C until use.

### LAMP reaction

**Figure 1:**
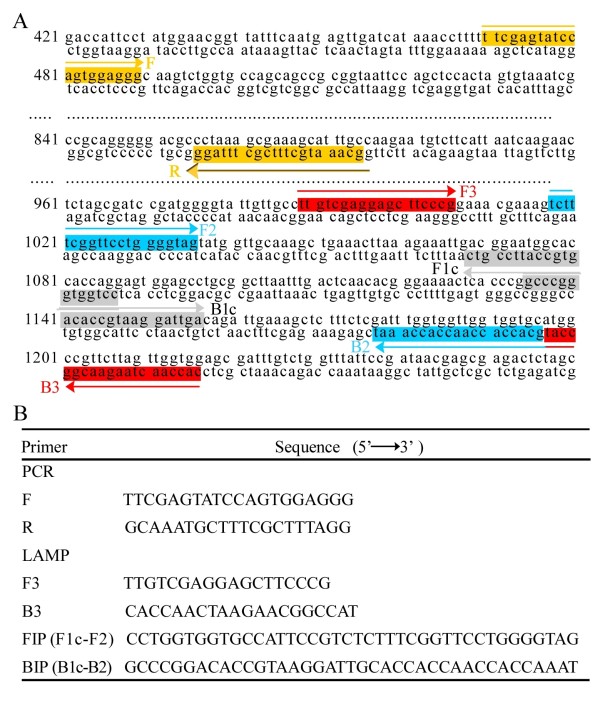
**Specific primers used in this study**. A: Position of primer sets in *A. cantonensis *18S rRNA gene for LAMP and PCR assay. B: Specific primers for the LAMP and PCR assay.

The LAMP reaction was conducted following methods described by Notomi, with minor modifications [30]. In brief, LAMP primers for the specific amplification of the *A. cantonensis *18S rRNA gene were designed according to sequence data in GenBank (accession number AY295804.1) using the Primer Explorer programme. The F3 and B3 primers were located outside of the two primers (FIP and BIP) in the *A. cantonensis *18S rRNA gene region. The sequences of the primers are shown in Figure 1. The LAMP reaction was performed according to the manufacturer's instructions (Eiken Chemical Co.Ltd; Tokyo, Japan). The LAMP reaction mixtures (final volume, 25 μl) contained 0.2 μM primers F3, B3 and 1.6 μM FIB, BIP. The other components of the reaction mixture were: 2.5 μl of 10× *Bst *DNA polymerase reaction buffer, 1 μl of 8 U/μl *Bst *DNA polymerase, 7 mM MgSO_4 _(2 μl), 5 μl of Betaine, and 1 μl of target sample. The LAMP reaction mixture was incubated at a range of temperatures (61, 63, 65, 67 and 69°C) for different times (30, 40, 50 and 60 min) to optimize the reaction conditions. The reaction was terminated by incubation at 95°C for 2 min.

### LAMP product detection

As part of the assay development, the LAMP products were initially detected by several methods, including electrophoresis in 1.5% agarose gels that were stained with GelRed™ (Biotium Inc.; Hayward, CA, USA) and photographed using a BIORAD gel system (Bio-Rad Laboratories; Hercules, CA, USA). A DL 2,000™ DNA Marker (Takara; Dalian, P.R.China) was used in electrophoresis to determine molecular weights. In addition, the products were visually detected by a colour change after the addition of SYBR green I dye to the tubes. One microliter of SYBR green I dye (100×) (Cambrex BioSciences Inc.; Rockland, ME, USA) was added to each tube containing LAMP products.

### Sensitivity and specificity of LAMP

The sensitivity of the LAMP assay was determined using *A. cantonensis *DNA samples and nine infected snail samples. A serial dilution of *A. cantonensis *genomic DNA was used at concentrations from 0.01 fg to 100 pg. The purified DNA was dissolved in 60 μl of double-distilled water, and 1 μl of the solution was used as the template for LAMP. The specificity of LAMP assay was tested using DNA samples from *T. gondii, P. falciparum, S. japonicum, C. sinensis, P. westermani *and *Anisakis*.

### PCR reaction

To compare and evaluate the sensitivity and efficiency of the LAMP protocol with the classical PCR technique, a simple PCR was conducted targeting the same gene using the same samples as for LAMP assay. The PCR reaction was performed following methods described by Qvarnstrom Y, with minor modifications [18]. A 405-bp DNA fragment of the *A. cantonensis *18S rRNA gene was amplified with sense primer (F, 5'-TTCGAGTATCCAGTGGAG GG-3') and antisense primer (R, 5'-GCAAATGCTTTCGCTTTAGG -3'). The PCR products were purified and sequenced.

## Results

### Development of an 18s rRNA-based LAMP assay

**Figure 2:**
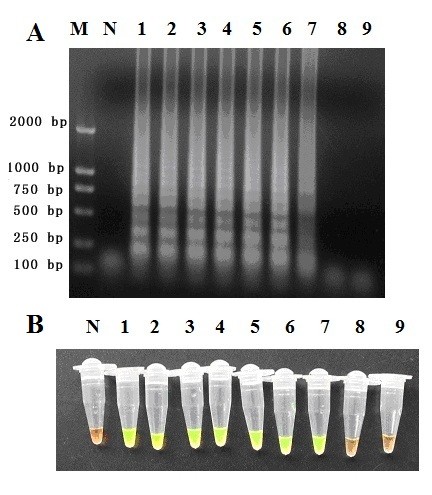
**Sensitivity of the LAMP assay for *A. cantonensis *detection**. LAMP reactions were performed with various concentrations of *A. cantonensis *DNA. lane 1, 1000 pg; lane 2, 100 pg; lane 3, 10 pg; lane 4, 1 pg; lane 5, 100 fg; lane 6, 10 fg; lane 7, 1 fg; lane 8, 0.1 fg; lane 9, 0.01 fg; lane M, DL2000 DNA Marker; lane N, negative control.

Agarose gel electrophoresis of all positive LAMP reactions revealed a ladder pattern with many bands of different sizes (Figure 2A), indicating that stem-loop DNA with inverted repeats of the target sequence was produced. In addition to gel electrophoresis, we used other methods to detect a positive reaction. Addition of SYBR green I dye to tubes after the LAMP reaction changed the colour of a positive reaction to yellowish green (Figure 2B, tube 1-7), whereas the negative reactions remained reddish orange (Figure 2B, tube N). All samples that were positive as determined by gel electrophoresis were also positive as determined by visual detection of colour change. The effect of LAMP amplification with different temperatures and periods of time was observed by gel electrophoresis to determine the optimum reaction condition. The best results were obtained when the reaction was incubated at a temperature of 65°C for 60 min (Data not shown).

### Sensitivity and specificity

**Figure 3:**
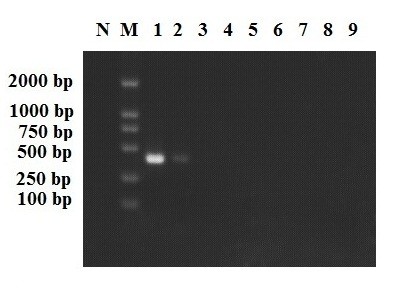
**Sensitivity of PCR for *A. cantonensis *detection**. PCR reactions were performed with various concentrations of *A. cantonensis *DNA. lane 1, 1000 pg; lane 2, 100 pg; lane 3, 10 pg; lane 4, 1 pg; lane 5, 100 fg; lane 6, 10 fg; lane 7, 1 fg; lane 8, 0.1 fg; lane 9, 0.01 fg; lane M, DL2000 DNA Marker; lane N, negative control.

**Figure 4:**
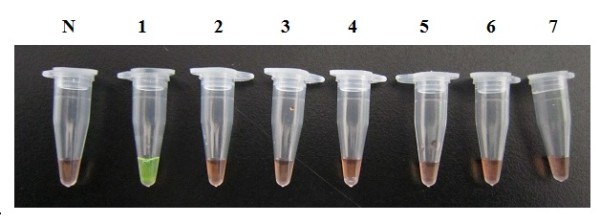
**Specificity of the LAMP assay for *A. cantonensis *detection**. Detection of DNA from *A. cantonensis *and other parasites by LAMP. LAMP reaction tubes were inspected visually. Positive reactions turned green after the addition of SYBR green I. Tube N, negative control; tube 1, *A. cantonensis*; tube 2, *T. gondii*; tube 3, *P. falciparum*; tube 4, *S. japonicum*; tube 5, *C. sinensis*; tube 6, *P. westermani*; tube 7, *Anisakis*

The sensitivity of the LAMP and PCR reaction was determined using 10× serial dilutions of *A. cantonensis *genomic DNA. The PCR detection limit was 100 pg (Figure 3), similar to the paper published before [12,31,32], while the LAMP detection limit was 1 fg (Figure 2), which shows that the LAMP assay is more sensitive than the PCR method. There is no cross-reaction with *T. gondii, P. falciparum, S. japonicum, C. sinensis, P. westermani *and *Anisakis *genomic DNA (Figure 4).

### LAMP and PCR assay of infected snails

**Figure 5:**
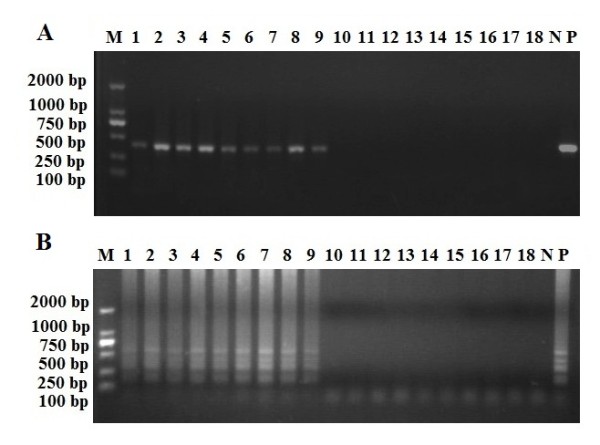
**PCR and LAMP amplification results from artificially infected snails**. A: Detection of *A. cantonensis *in snail samples by PCR. B: Detection of *A. cantonensis *in snail samples by LAMP. lane M, DL2000 DNA Marker; lane P, positive control; lane N, negative control; lanes 1-9, infected snails; lanes 10-18, uninfected snails.

Nine samples from artificially infected snails were verified using microscopic examination, then analyzed using the LAMP and PCR method. The sequence results of PCR products demonstrate specific *A. cantonensis *gene was amplified. All these samples were positive for *A. cantonensis*, while the samples from uninfected snails remained negative (Figure 5), which shows that LAMP is sensitive and effective for diagnosis of *A. cantonensis*.

In conclusion, the detection limit of the LAMP assay was 1 fg of *A. cantonensis *genomic DNA, while the detection limit of the PCR is 100 pg, which shows that LAMP assay is more sensitive than PCR method. The specificity of the LAMP assay was confirmed using DNA extracted from *T. gondii, P. falciparum, S. japonicum, C. sinensis, P. westermani *and *Anisakis*, and no cross-reactivity was found. Nine samples from artificially infected snails determined by the microscopic examination were analyzed using the LAMP and PCR method. All these samples were positive for *A. cantonensis*, while samples from uninfected snails remained negative, which shows that LAMP is sensitive and effective for diagnosis. Thus, it was demonstrated that the LAMP technique could be used to amplify *A. cantonensis *DNA with high specificity and efficiency.

In recent years, the detection of snails by PCR has become an important tool for surveillance of *A. cantonensis*. Despite the clinical utility of PCR-based techniques, they have the inherent disadvantage of being time-consuming as well as requiring special equipment which is not suitable for field application. We demonstrate in this study that the LAMP technique can be used to amplify *A. cantonensis *DNA under isothermal conditions with high specificity and efficiency. It has a minimal equipment requirement and can be accomplished in one hour or less. The specificity and sensitivity of the LAMP assay shows that LAMP is effective for diagnosis of *A. cantonensis *and is suitable for large-scale field surveys.

## Competing interests

The authors declare that they have no competing interests.

## Authors' contributions

CR conceived the study, performed the main experiments, and wrote the manuscript. TQB assisted with the LAMP and PCR reaction. ZY and LvS established the snail infection model. LD, ZMM and KQM prepared parasites and microscopic examination. LSH conceived and supervised the study. All authors read and approved the final manuscript.
